# In silico prediction of cancer immunogens: current state of the art

**DOI:** 10.1186/s12865-018-0248-x

**Published:** 2018-03-15

**Authors:** Irini A. Doytchinova, Darren R. Flower

**Affiliations:** 1 Faculty of Pharmacy, Medical University of Sofia, 2 Dunav st, 1000 Sofia, Bulgaria; 2 School of Life and Health Sciences, Aston University, Aston Triangle, Birmingham, B4 7ET UK

**Keywords:** Cancer immunogens, Databases of cancer immunogens, Prediction of cancer immunogens, Dendritic cell-based vaccines, Multi-epitope vaccines

## Abstract

Cancer kills 8 million annually worldwide. Although survival rates in prevalent cancers continue to increase, many cancers have no effective treatment, prompting the search for new and improved protocols. Immunotherapy is a new and exciting addition to the anti-cancer arsenal. The successful and accurate identification of aberrant host proteins acting as antigens for vaccination and immunotherapy is a key aspiration for both experimental and computational research. Here we describe key elements of *in silico* prediction, including databases of cancer antigens and bleeding-edge methodology for their prediction. We also highlight the role dendritic cell vaccines can play and how they can act as delivery mechanisms for epitope ensemble vaccines. Immunoinformatics can help streamline the discovery and utility of Cancer Immunogens.

## Background

Cancer is a catch-all term for a constellation of diseases typically characterised by abnormal cell division. The term cancer can be traced to the Greek physician Hippocrates (460-370 BC), who used the terms *carcinoma* and *carcinos* to refer to ulcer-forming tumours and non-ulcer forming tumours. In Greek, these words refer to a crab. The Roman physician, Celsus (28-50 BC), translated this to cancer, the Latin for crab. Galen (130-200 AD) used the Greek word *oncos*, meaning swelling to describe tumours. Almost all cells and tissues can become cancerous, but fortunately most cancers are very rare. Yet cancer remains one of the prime health issues of our time [1].

In 2012, there were about 14 million new cancer cases worldwide and 8.2 million deaths. Deaths caused by cancer is very high in developed countries [1]. In 2014, the US recorded 591,700 deaths from cancer, with approximately 197,233 deaths in women and 394,466 deaths in men; about 22% of all deaths. The equivalent UK figures were 163,000 deaths, or 450 deaths per day; with approximately 86,500 cancer deaths in men and 76,900 deaths in women; about 25% of all deaths. Yet over half of the global cancer burden occurs in less well developed countries. Lung, bowel, liver, and stomach, are the commonest cancers globally, equating to 4 in 10 deaths worldwide. At about 1 in 10 cases, smoking-related lung cancer is the commonest male cancer.

A cancer can be classed as either “common” or “rare” based on relative prevalence. The precise threshold between classes remains open. The US National Cancer Institute (NCI) identifies “rare” as those cancers with a prevalence below 15 in 100,000 [2]. This means only 11 adult cancers are defined as common in the US: prostate, breast, lung, bowl, cervical, bladder, rectum, ovary, kidney, melanoma, and non-Hodgkin lymphoma [3]. Other adult cancers - about 25% of all adult cancers - are, by this definition, “rare” [3].

Driven by the financial exigencies governing drug discovery and development, effective cancer treatment is significantly skewed towards common cancers. As an example, there are over 20 Category 1 intervention - uniform consensus that intervention is appropriate and based on significant evidence - for prostate and breast cancer, the commonest cancers in men and women [4–6]. Yet none exist for say the bone cancers, chondrosarcoma or chordoma, which affect under 1000 individuals annually in the US [7–9].

Survival varies considerably between different cancers. It ranges from 98% for testicular cancer to about 1% for pancreatic cancer. Most common cancers have a 10-year survival above 50%. Over 80% of those with cancers which are easy to treat and/or diagnose survive for 10+ years, yet less than 1 in 5 people with hard-to-treat or hard-to-diagnose cancers survive for 10 years or more [10]. Thus cancer remains a pivotal unmet medical need, driving both technical innovations and improved clinical practice, resulting in dramatic improvement in cancer treatment. In the UK, mortality rates peaked in the 1980s, with overall cancer mortality falling by 14% since the early 1970s, with a 22% decrease in men and an 8% decrease in women. In the UK, mortality for all cancers is predicted to decrease by 15% in the period 2014-2035, reaching less than 280 deaths per 1 hundred thousand by the year 2035 [10].

According to somatic mutation theory, mutations in DNA and epi-mutations disrupt the programmed regulation of cell division, upsetting the balance between proliferation and apoptotic cell death, resulting in excessive and uncontrolled division. Many mutations lead to cancer, but most do not. The treatment of solid tumours in particular has changed dramatically in recent years due to enhanced molecular diagnostics helping to identify a burdening number of addressable oncogenic abnormalities including in-frame insertions/deletions and amplification or rearrangements and gene activating point mutations.

Historically, cancer has been treated by small molecule drugs. A number of anti-cancer drugs are classed as agents of so-called chemotherapy. These are typically characterised by significant side-effects, as many affect cells indiscriminately. The main types of chemotherapy include DNA-damaging alkylating agents, including structurally-simple reactive molecules such as Busulfan; Antimetabolites, which compete with natural nucleotides for incorporation into DNA or RNA, impairing DNA replication, such as 5-fluorouracil; Anti-tumour antibiotics, such as complex natural product Epirubicin; Topoisomerase inhibitors, which interfere with DNA unzipping prior to replication, such as Topotecan; Mitotic inhibitors, such as plant-derived natural product Paclitaxel; and Corticosteroids, such as Prednisone [11]. Other, more targeted therapies are now appearing. Precision medicine can be defined as therapy individualised to each tumour, achieving this by exploiting quantifiable genetic alterations as de fact predictive biomarkers and/or as therapeutic or prophylactic targets for the next generation of cancer treatments.

Most recently, immune based approaches have gained significant saliency. Immunotherapy directed against cancer, include a triumvirate of main approaches: monoclonal antibodies, immune checkpoint inhibitors, and vaccines. The immune response has two arms: the humoral, or antibody-mediated, arm and the cellular arm, mediated primarily by T cells. Historically, almost all vaccine prophylactic responses have been mediated by Antibodies. Each human has billions of potential antibodies capable of recognizing proteins and tagging them for elimination. The individual ‘baseline’ for addressing antigen challenge is the primary naïve antibody repertoire. The structural and sequence diversity of this baseline enables the immune system to recognize, at least weakly, a very large set of antigens. Unfortunately, only a subset of Tumour Associated Antigens (TAAs) are amendable to the antibody mediated responses necessitating the exploration of cellular immune mechanisms as a replacement or adjunct therapy.

The effectiveness of potential therapeutic cancer vaccines is often reduced by mechanisms in cancer patients that suppress T-cells and antigen presenting cells (APCs). Most cancer vaccines induce anti-tumour immune responses when formulated with strong adjuvants, due to the general lack of immunogenicity exhibited by vaccines not derived from whole pathogens. Vaccination against cancer takes several forms: DNA-based vaccines, RNA-based vaccines, and DC-based vaccines.

DNA vaccines: trials to evaluate the efficacy of Inovio Pharmaceuticals combination vaccine INO-3112 are planned against cervical, head, and neck cancers (NCT02172911, NCT02163057) “http://ir.inovio.com/news-and-media/news/press-release-details/2017/Inovio-Begins-Phase-3-Clinical-Trial-of-VGX-3100-for-the-Treatment-of-HPV-Related-Cervical-Pre-Cancer/default.aspx”. INO-3112 contains plasmids encoding E6 and E7 (VGX-3100) [12] combined with DNA-based IL12 delivery (INO-9012). Inovio’s preventive anti-HIV DNA vaccine, PENNVAX-G, used in a prime-boost protocol with altered pox virus vector, has a satisfactory safety and immunogenicity profile [13]. This study should foment design of anti-cancer therapeutic vaccines by exploring prime-boost regimens using DNA vaccines and viral boosts. The Vaccibody-developed DNA-based vaccine VB10.16 targets HPV16 “http://www.vaccibody.com/vb10-16/”. A trial (NCT02529930) is set to launch; if successful it should provide an innovative and much needed non-invasive way to treat HPV-induced cervical cancers.

RNA vaccines: Sahin’s group pioneered use of lipid-based positively-charged nanoparticles delivering RNA encoding TAAs, to target DCs in vivo and thus simulate an anti-viral response [14]. This is currently undergoing a phase I trial in melanoma patients (NCT02410733). A two component RNA vaccine platforms launched by Curevac has also yielded promising results in early trials (NCT00923312) [15].

DC-based vaccines: multiple platforms are being developed to harness ex vivo activated DC vaccines for cancer immunotherapy. These platforms include the with-antigen loading vaccine DCVax-Direct “https://www.nwbio.com/dcvax-direct/” and the without-loading vaccine DCVaxL “https://www.nwbio.com/dcvax-technology/”. Similarly, the Individualized Vaccines Against Cancer (IVAC) platform uses autologous DCs loaded with individually sequenced neo-antigens (NCT02035956, NCT02316457). The potential of DC vaccines is only beginning to be explored.

Protein-based vaccines: As TAA are poorly immunogenic, an adjuvant able to generate effective immune response should be added in the protein-based vaccines [16, 17]. Aluminum salts (alum) are used as adjuvants promoting protective humoral immunity, while for the activation of cell-mediated immunity are used conserved moieties associated with pathogen or endogenous alarmins like head shock proteins (HSPs). HSPs are able to induce both innate and addaptive immune responses. The first autologous HPS vaccine, Oncophage, failed to demonstrate survival benefits in Stage IV melanoma patients although stage I and II patients seemed to benefit from vaccination [18]. Wang et al. [19] have developed a platform for generating of chaperone complexes between HSPs and clinically relevant TAA.

Computational prediction can give important insight into both antibody and cellular immune responses. Here we examine non-experimental approaches to the cataloguing and prediction of TAAs. We describe the classification of TAAs into separate categories, databases that curate and classify TAAs, servers that facilitate the accurate and robust prediction of TAAs, and the role of DC vaccines to fight cancer and deliver pre-loaded epitope ensemble vaccines.

### Classification of tumour antigens

Tumour Antigens are expressed largely, but not solely, by tumour cells. Utilisation of defined tumour antigens represents perhaps the most likely current approach accurately to directing immunotherapies towards differentiating cancer from neoplastic cells. As such, tumour antigens form the underpinning bedrock of modern tumour immunotherapy.

Tumour Antigens can be effectively classified using a scheme based primarily on their origin and distribution. Although there is no officially sanctioned classification system for tumour antigens, most experts in the field [20] broadly accept a classification protocol that makes use of the broadness of expression of individual antigens and how specific they are to a particular form of tumour. According to such a classification, tumour-associated antigens can be broadly divided into the following thematic categories:

1. Unique tumour-specific antigens (TSA). They occur within a single type of tumour in one patient. Such antigens can form excellent targets for personalized cancer immunotherapy. Examples include MAGE melanoma-associated genes.
2. Shared lineage-specific differentiated antigens. They are expressed in both tumor and healthy tissue and typically viewed as poorer or secondary targets for immunotherapy. However, CD19, a B cell marker, is one of the most successful cancer targets [21].
3. Shared tumour-specific antigens or cancer neo-antigens. They are expressed in different tumour but not in healthy tissues and can form the basis of ‘off-the-shelf’ vaccines applicable in a broad array of cancers and patient populations. These are unique MHC restricted antigens created by mutations in tumour cells. Vaccines designed to target these antigens should theoretically be able to target tumour cells specifically while obviating the induction of general autoimmunity or tolerance. However, not all tumours express immunogenic neo-antigens. Moreover, tumours and patients have unique neo-antigen repertoires necessitating personalized neo-antigen discovery programs that facilitate the development of personalized vaccines against predicted neo-antigen epitopes.
4. Shared over-expressed antigens. They are not tumour-specific but have a much greater expression in tumours compared to neoplastic cells. This category covers antigens that are present in both normal and tumour cells but which are substantially over-expressed by tumour cells. Example antigens falling into this category include Her2/Neu [22], mesothelin [23], lineage and tissue restricted differentiation antigens such as melanoma differentiation antigens (Tyrosinase Related Protein-2 and Melan-A (MART-1)) and Oncofetal antigens (Carcinoembryonic antigen) [24].
5. Oncoviral Antigens: These are antigens expressed by viruses, like human papilloma virus (HPV) and Merkel cell polyomavirus that cause tumorigenic transformation in cells. As these antigens are typically only found expressed on infected cells, they are able to be recognized by the immune system as ‘non-self’ distinct from the “self” or host protein [25].

As is made evident by the above classification, not all TAA are suitable for cancer immunotherapy. According to Kessler and Melief [20], a TAA could be considered as a potential cancer immunogen, if it responds to the following criteria: to be tumour-specific and widely shared, to play a role in the oncogenic process, or to promote cancer cell survival and thus provoke an immune response. It is possible, at least theoretically, to target TAAs using either an antibody or a cellular approach, although in practice this depends on the level and time-course of antigen expression. Antigens selectively expressed on the cell surface either constitutively or for periods of long duration are potent targets for antibodies, but antigens that only appear on the surface as epitopes bound to MHCs are clearly only amenable to surveillance by cellular immunity.

### Databases of cancer immunogens

Due to the very extensive and intensive research efforts focussing on cancer aetiology and therapy seen during the last few decades, a plethora of cancer-associated data has accumulated and has subsequently been archived in a wide variety of different databases and repositories [26]. Here, we review only the most relevant databases for cancer immunogens available free on the web:

1. The Peptide Database of the Cancer Research Institute [27] has been established in 2001 and today it comprises more than 400 fully validated tumour antigenic peptides (URL: https://www.cancerresearch.org/scientists/events-and-resources/peptide-database). They are classified as mutated, tumour-specific, differentiated, and overexpressed. Other antigens are classed as potential, as a catch-all for those antigens whose comprehensive characterization is not yet reported.
2. The database of differentially expressed proteins (or dbDEPC) contains 4029 differentially expressed proteins, collected from 331 mass spectrometry experiments across 20 types of human cancer [28, 29]. This database allows one o search for proteins undergoing changes in certain cancers, shows protein expression heat-maps across various cancers, and relates protein expression changes to changes at the genetic level. Moreover, it also includes information on experimental methodology used, sophisticated tools for filtering user-specified analysis, and a tool for analysing networks.
3. The Cancer-Testis database (CTdatabase; URL: http://www.cta.lncc.br/) contains known cancer testis antigens, typically proteins of known immunogenicity differentially expressed by different forms of cancer versus normal tissue [30]. The database contains links to relevant CT antigen articles plus basic information such as gene names, their aliases, genomic location and corresponding RefSeq accession numbers, known splice variants, reported gene duplications, mRNA levels in cancer and normal tissues, as well as antigen-specific immunological responses in cancer patients.
4. TANTIGEN (URL: http://cvc.dfci.harvard.edu/tadb/) is a database housing a comprehensive collection of cancer antigens, with over 1000 measured tumour peptides from 368 proteins [31]. TANTIGEN is thus a rich data source for those working to discover tumour-associated epitopes and neo-epitopes. Archived peptides are classified in a set of categories:
  A. Peptides which bind in vitro to HLA but are not reported to engender in vivo or in vitro cell responses.
  B. Peptides found to bind HLA and to engender an in vitro T cell response.
  C. Peptides shown to mediate in vivo tumour rejection.
  D. Peptides naturally processed and presented, as identified by physical techniques.

### Servers for prediction of cancer immunogens

As both CD8+ and CD4+ T cells play a significant role in tumour rejection, most of the in silico methods for cancer immunogens prediction utilize servers for T-cell epitope prediction. Cancer immunogens are processed mainly in the dendritic cells by a cascade of enzymatic digestion in proteasomes or endosomes followed by assembling with HLA class I or class II proteins in the endoplasmic reticulum and presentation of the complexes on the cell surface where they are recognized by the CD8+ and CD4+ T cells, respectively [21]. The servers for T cell prediction utilize a wide range of different algorithms for prediction of peptide binding to HLA class I and class II proteins [32–34]. Servers trained to recognize whole cancer immunogens include:

1. VaxiJen was the first server for prediction of cancer immunogens applying a unique alignment-free algorithm [35]. The hydrophobicity, molecular size and polarity of amino acid residues were presented by *z*-scores [36]. The strings were converted into uniform vectors by auto- and cross covariance (ACC) transformation [37]. The algorithm was trained on a set of 75 known tumour antigens and 75 randomly chosen human proteins and tested on a set of 25 known tumour antigens and 25 human proteins. VaxiJen identified 96% of the test tumour antigens and 76% of the test human proteins with overall accuracy of 86% at threshold of 0.5.
2. TIminer (Tumor Immunology miner) is a pipeline for mining tumour-immune cell interactions from next-generation sequencing data [38]. It provides HLA class I typing by RNA-seq, characterization of immune infiltrates and quantification of tumour immunogenicity through immunophenogram and immunophenoscore, and neoantigen prediction from mutated proteins binding to patient-specific HLA class I proteins.
3. MuPeXI (mutant peptide extractor and informer) identifies tumour-specific peptides and assess their potential to be neo-epitopes [39]. It consists of several steps: identifies protein sequence changes that result from a genomic alteration, retains the alteration-containing peptides as potential neo-peptides, compares them to the human proteome and penalizes the identical as non-immunogenic, predicts the binding affinities of neo-peptides to patient-specific HLA types, and prioritize the neo-peptides which are likely to be abundantly presented by patient’s HLA and recognized by the T cells.

To improve these servers, we need both an improvement to the underlying data – in terms of quantity and quality - and to the breadth and robustness of algorithms. What is also very much required is a much better and much more carefully constructed tranche of negative training sets and algorithmic learning protocols over and above just simple improvements in reported accuracy. We should balance the selection of negative test sets so that any signal present reflects antigenicity and no other quality, selecting similar origin species, similar subcellular locations, similar protein lengths, and similar functions. Robustness in particular is seldom addressed by method developers. An over-specified algorithm which works well interpolating within a poorly-defined multidimensional subset of the overall chemical space is seldom likely to extrapolate well to unseen data that clearly lies outside such a space.

### Antigen selection for cell-based cancer treatment: subunit and epitope ensemble vaccines delivered by dendritic cell and antigen selection for CAR T-cell therapy

Several decades ago, the advent of biologics revolutionized the pharmaceutical industry. Today, biomedicine is on the cusp of another revolution: cells as therapies. The potential of such novel therapies is enormous but significant challenges remain. Natural in origin or designed, such cells will present problems scientific, regulatory, and economic in nature. Cellular medicines will necessitate the development of a foundational cellular engineering science providing a systematic framework for the safe and predictable modulation of cell behaviour. In the vanguard of cellular medicine is the development of DC-based vaccines and the advent of CAR T-cell therapy. It should be noted that the immunoinformatic prediction of cancer antigens, as adumbrated in preceding sections, potentially underpins several important therapeutic strategies - CAR T-cell therapy and DC vaccines – as well as epitope ensemble vaccines. We explore these exciting strategies here.

Amongst all APCs, so-called dendritic cells (DCs), have the greatest perceived capacity to initiate innate and adaptive immune responses. DC based vaccines offer the potential therapeutic benefits of suppressive therapies against pathogens, tumours, and/or autoimmune diseases [40]. Consequently, there has been a maelstrom of activity in creating and testing DC cancer immunotherapy. DC vaccines are primarily used to treat cancer. For example, sipuleucel-T is a US approved DC-based vaccine for treatment of hormone-insensitive prostate cancer.

In the 1970’s, Ralph Steinman discovered DCs in the spleen. Post 1970’s, it was revealed that DCs exist in non-lymphoid and lymphoid tissues as antigen presenting cells. The theoretical framework was based on Daniel Hawiger’s experiment which utilised antigens specific for diseases such as: tuberculosis, diabetes, HIV, allergy or cancer. The specific antibody was used as a delivery vehicle and carried these antigens to DCs. This notion was applied by Steinman, exploiting varying receptors to trigger an immune response by targeting DCs [41].

DCs are present in an immature state in the blood, upon activation they migrate to the lymph tissue where they network with B cells and T cells. Immature DCs migrate through the blood stream from the bone marrow to enter tissues, ingesting particulate matter by phagocytosis and persistently absorb large amounts of extracellular fluid by micropinocytosis. Also presenting where there is contact with the external environment as they are portals of entry for infectious organisms, including the lining of the nose, lungs, intestine and stomach. DCs take up and process antigens and migrate to regional lymph nodes.

Manipulation of the immune system to eliminate cancer cells has long been a clinical and preclinical focus. Although achieving some success with cytokines such as IFN-γ and IL-2, an immunotherapy with proven clinical outcomes remain elusive. As previously, peptide-based approaches were discouraging, isolating stem cells from cultured blood resulted in sipuleucel-T (Provenge). Stem cells were loaded with cancer antigens and became sensitised. Sensitised DCs are injected into the skin and travel to the lymph node where they seek out specific lymphocytes. The DCs then initiate specific lymphocytes to multiply and attack cancer cells [42].

Thus the secret to future effective DC-based vaccines capable of combatting cancer is the identification of potent cancer antigens. A key alternative to whole protein immunogens is the idea of loading DCs with an epitope ensemble vaccine as a prelude to creating an anti-cancer vaccine. Here immunoinformatics can help.

Efforts supporting the development of a T-cell poly-epitope or epitope ensemble vaccine fall into two camps: un-validated prediction-only methods that predict supposedly high-binding epitopes [43] and more modern approaches that use immunoinformatics to select rather than predict the best epitopes suitable for forming a vaccine [44, 45]. Both rely on the development of accurate, reliable, and robust algorithms for the prediction of epitope affinity [46] and processing [47]. Here accurate refers to the nearness of results to reality, reliable – to the broadness of this accuracy in terms of distinct epitopes and MHC alleles, and robust – to the ability to deal with new data radically different from that it has seen before. Most algorithms, show variable performance in regard to these different criteria.

Prior to DC-based vaccines, small-molecule based chemotherapy and other toxic therapies were used to prevent or slow the progression of tumours. DC-based vaccines have the ability to initiate an immunological response that will hinder the development of malignancies even whilst the cancer cells mutate, and thus represent a potential step-change in cancer treatment. DC vaccine studies have shown that stimulating antigen specific cytotoxicity in vivo and in vitro exhibit a lack of toxicity and increase survival rates. In 16 different clinical trials, over 200 patients were treated for brain tumours, and have proven to treat metastasis although the clinical response is seemingly dependent on when immunotherapy is administered. Patients who benefit most are patients in early stage metastasis with a lower tumour burden. Multiple vaccines rather than a single vaccine stimulate a more multivalent response.

Currently, most DC therapies are rather limited in their scope, since they are typically used as part of a complex combination treatment rather than a monotherapy. Nonetheless, current state-of-the-art DC-based therapies is the cause for much optimism since they are clearly a prime candidate for future elaboration, leading to a wealth of promising future treatments.

Recently, immunotherapy, rather than vaccination per se, has the potential “fifth pillar” of cancer treatment. So-called Adoptive Cell Transfer, or ACT, collects patients’ immune cells to treat cancer; of the various types of ACT, Chimeric antigen receptors (CARs) T-cells seems the most promising. When a CAR is derived from an antibody, the resulting T-cell will combine its own effector functions with an antibody’s ability to recognize non-protein antigens and be freed from obligatory major histocompatibility complex restriction.

Hitherto, CAR T-cell therapy has been limited to small-scale clinical trials, mostly in blood cancer patients. In 2017, two CAR T-cell therapies gained approval by the Food and Drug Administration (FDA): one for patients with advanced lymphomas, the other for acute paediatric lymphoblastic leukemia. Yet this is still an early phase for CAR T-cell therapy, with questions over their potential effectiveness against solid tumours. In particular, technical questions about the identification and selection of appropriate antigens for incorporation into CARs remain.

To a crude, first approximation, a CAR is composed of an extracellular targeting domain (ectodomain), and transmembrane region, and an intracellular T-cell signalling domain (endodomain) [48]. The ectodomain can constructed from a limited repertoire of signalling domains, such as ZAP70 or CD28. The ectodomain is a more challenging design puzzle, as it is exquisitely linked to the form of cancer being targeted. While immunoglobulin domains in their antibody and TCR guises are perhaps the most obvious candidates, a plethora of ever-increasing number and diversity continue to emerge [49, 50]. These include, inter alia, adnectins, Affibodies, Avimers, DARPIns, Fynomers, Kunitz domains, knottins, and Nanobodies. The challenge here is twofold: one predicting using VaxiJen or equivalent approach the appropriate target.

However, perhaps the most interesting, intriguing, and exciting alternative is the possibility of including anticalins [51, 52] as antibody surrogates. Anticalins are non-natural engineered lipocalins able to bind small molecules in a hapten-dependent but conjugate antigen-independent manner. This would open up metabolites secreted in a cancer-dependent fashion by tumours as putative targets for anti-cancer CAR T-cells. Moreover, lipocalins as well as binding small molecule ligands of all kinds, also have the capacity to bind macromolecules with high specificity [51]. This could open the way to dual specificity anticalin CAR T-cells able to bind both cancer-specific metabolites and cell surface receptors, enlarging the homing capacity and cell-targeting abilities native to T cells.

## Discussion

The worth, value, and utility of vaccines, though clear for all to see, is not yet unchallenged; yet most reasonable people are likely to agree that they are, qualifications apart, a thing of inestimable value and utility. Existing vaccines are not perfect. One might argue that their intrinsic complexity, and the highly empirical nature of their discovery over decades, and the fraught nature of their manufacture, is a root of current mistrust. In some senses this also hampered the progress of cancer vaccines and immunotherapy. Finally, these are beginning to make some headway.

Computational prediction has a part to play, one of the strongest messages to emerge from this review is that immunogenicity is a multi-factorial property: some protein antigens are immunogenic for one reason, or set of reasons, while another protein will be immunogenic for another possibly-tangential reason or set of reasons. Each such a causal manifold seems dauntingly complex and confusing. The prediction of immunogenicity for cancer antigens is a greater problems still in multi-factorial prediction since we must factor in the high degree of antigenic similarity to other host proteins. Thus the search for new antigens is a search through a multi-factorial landscape of contingent causes. As noted above, the immunoinformatic prediction of cancer antigens potentially underpins several important therapeutic strategies, including epitope ensemble vaccines, CAR T-cell therapy, and DC vaccines.

To develop proper predictive approaches to the prediction of cancer and other immunogenic antigens we need to address several issues. We require more “positive” and carefully curated, validated data focussed on cancer. While there are databases of vaccine antigens - AntigenDB [53] is a dedicated resource directly addressing this, as well as IEDB [54] - similar yet better data resources are still required, suggesting the need to enlarge, deepen, and broaden available data collections. We also require much better and much deeper representations of the sequence data. Single descriptors characterising the whole sequence [55], and other multivariate descriptors of sequences. One could envisage a phase space of disjoint descriptor variables from which variable selection protocols could extract a compact, near-optimal choice of indicative variables. Also, better algorithms are needed. Powerful machine learning toolkits, such as Weka, are already available, and these are more than capable of delivering robust and extensible methods provided the data and the data representation are adequate. Yet, as new algorithms appear we must not be complacent but open, embracing proven innovations.

Better protocols for establishing the immunogenicity of identified potential vaccines are desperately needed. This work is that of the experimentalist. Here a fast, straightforward methodology is required which projects a more consistent, clearer, and much more accurate picture of the immunogenicity of individual proteins. As with many computational studies of real world problems, there is also general need for experiments able to validate predictions. The *in silico* analyses of pathogen genomes and virtual proteomes, has led to the publication of innumerable papers reporting potential but unverified vaccine candidates [56–58]. Such papers typically use methodology largely embodied in web-servers: operating such systems is facile, and the resulting analysis straightforward. Publishing unverified papers ultimately becomes counterproductive. Science progresses through independent corroboration by verification by peers. Science progresses faster when people do not waste time on fruitless research. Many are rightly alarmed by the increasing perception that the complex results of present day science cannot be reproduced and validated. Explanations are legion, including increased levels of scrutiny and institutional pressure on research and individual researchers. Arguably, the greatest issues are the increasing complexity and instrumentality of modern experimentation, in the opaqueness of many systems being studied, and the daunting technicality of analysing and teasing out the nature of many experiments. Computational experiments may be reproducible in themselves but without robust and reproducible experimental validation mean little. Other vaccine prediction studies give credibility to their results [59, 60] by linking vaccine design to experimental validation. Even in the current atmosphere of hysteria and hyperbole over AI, prediction lacking validation exerts slight influence and convinces few.

## Conclusions

The utility of vaccines, though clear to most of us, is not yet unchallenged. Existing vaccines are not perfect. This also hampered cancer vaccines and immunotherapy. Finally, these are beginning to make some headway. Computational prediction has a part to play, one of the strongest messages to emerge from this review is that immunogenicity is a multi-factorial property. The prediction of immunogenicity for cancer antigens is a greater problems still in multi-factorial prediction since we must factor in the high degree of antigenic similarity to other host proteins. Immunoinformatics is poised to deliver on its potential and open up a whole new era in Cancer immunotherapy.

## Abbreviations

AI: Artificial intelligence
APC: Antigen presenting cell
DC: Dendritic Cell
DNA: Deoxyribose nucleic acid
IEDB: Immune epitope database
RNA: Ribonucleic acid
TAA: Tumour associated antigen
TSA: Tumour specific antigen
